# Bimodal electroencephalography-functional magnetic resonance imaging dataset for inner-speech recognition

**DOI:** 10.1038/s41597-023-02286-w

**Published:** 2023-06-13

**Authors:** Foteini Simistira Liwicki, Vibha Gupta, Rajkumar Saini, Kanjar De, Nosheen Abid, Sumit Rakesh, Scott Wellington, Holly Wilson, Marcus Liwicki, Johan Eriksson

**Affiliations:** 1 Luleå University of Technology, Department of Computer Science, Electrical and Space Engineering, Embedded Intelligent Systems LAB, Luleå, Sweden; 2 University of Bath, Department of Computer Science, Bath, UK; 3 Umeå University, Department of Integrative Medical Biology (IMB) and Umeå Center for Functional Brain Imaging (UFBI), Umeå, Sweden

**Keywords:** Databases, Cognitive neuroscience

## Abstract

The recognition of inner speech, which could give a ‘voice’ to patients that have no ability to speak or move, is a challenge for brain-computer interfaces (BCIs). A shortcoming of the available datasets is that they do not combine modalities to increase the performance of inner speech recognition. Multimodal datasets of brain data enable the fusion of neuroimaging modalities with complimentary properties, such as the high spatial resolution of functional magnetic resonance imaging (fMRI) and the temporal resolution of electroencephalography (EEG), and therefore are promising for decoding inner speech. This paper presents the first publicly available bimodal dataset containing EEG and fMRI data acquired nonsimultaneously during inner-speech production. Data were obtained from four healthy, right-handed participants during an inner-speech task with words in either a social or numerical category. Each of the 8-word stimuli were assessed with 40 trials, resulting in 320 trials in each modality for each participant. The aim of this work is to provide a publicly available bimodal dataset on inner speech, contributing towards speech prostheses.

## Background & Summary

Although research in the field of brain-computer interfaces (BCIs) began in the 1960s, it has accelerated in recent years due to advances in machine learning, imaging, and other data collection modalities^1,2^. A core aim of BCI research is to assist people who have lost the ability to move, speak or communicate with their environment. Inner speech can be described as the inner voice inside our heads; this phenomenon is used when thinking without any accompanying muscle movement or speech articulation^3–6^.

A research area close to inner speech is the area of imagined speech^7–13^. Decoding inner speech from brain activity is a burgeoning research area and has applications for BCI paradigms such as speech prostheses^8,9^, in clinical contexts—for example, informing models of psychiatric disorders in which inner speech is disturbed (schizophrenia^14,15^)—and in neuroscience, by deepening our understanding of the spatiotemporal neural dynamics of inner speech^16^.

Preliminary results have revealed that the most important parts of the brain for inner speech are the frontal gyri, including Broca’s area, the supplementary motor area and the precentral gyrus^17,18^. Furthermore, core representations of the language system (phonology, lexicon, and syntax) have a clearly distinguishable spatial distribution in the neocortex^19–21^. This distribution of brain regions is remarkably similar across languages and across individuals^22^, regardless of why these language representations are accessed (for production or comprehension) or how they are accessed (visually (by reading) or auditorily (by listening)).

BCI technologies use brain data acquired by invasive (ECOG)^23^ or noninvasive modalities (e.g., electroencephalography (EEG)^24^, functional magnetic resonance imaging fMRI)^25^, functional near-infrared spectroscopy (fNIRS)^10,26^ and magnetoencephalography (MEG)^27,28^) to establish an interface between humans and machines; in particular EEG data are the most commonly used in BCIs; and fMRI is a typical complimentary modality due to high spatial resolution. BCI paradigms include motor imagery^29,30^ and external stimulation paradigms, such as the visual P300^31^. In motor imagery paradigms, patients imagine their movement without overtly performing the action; in the visual P300 paradigm, patients typically use the direction of their eye gaze to spell out words by selecting among flashing stimuli, again without additional overt movement, which requires substantial participant concentration and eye control^32^. In recent years, the research focus for BCIs used to enable Augmentative and Alternative Communications (AACs) has turned to inner speech.

Research on inner speech decoding has investigated the use of all invasive ECoG^33,34^ and noninvasive methods^11,35–38^. Various datasets have been acquired. Selected studies presenting EEG and fMRI are as follows: KARA ONE^12^ is a dataset of inner and outer speech recordings that combines a 62-channel EEG with facial and audio data. The dataset includes 12 participants, and the lexicon contains 7 phonemes and 4 phonetically-similar words for binary phonological classification. Coretto^13^ provided a dataset containing a 6-channel EEG recordings of inner and outer speech recordings of 5 vowels and 6 words. Nguyen^39^ generated a dataset that contains a 64-channel eeg recordings of inner speech from 15 subjects, with a lexicon of 3 vowels and 5 words (note that the work also introduces new algorithms, but this is secondary for our study). Ferreira^40^ provided a fMRI dataset of inner speech recordings from 20 native Portuguese speakers that consisted of cardinal vowels, monosyllabic and disyllabic words, and sentences. Recently, Nieto *et al*.^7^ published an open-source unimodal EEG dataset of inner-speech BCI commands in Spanish.

The main limitation of such unimodal datasets is a much lower bound for possible recognition performance, as either temporal or spatial aspects of the data are not included. Unimodal datasets based on either EEG or fMRI can have drawbacks with regard to their temporal and spatial resolutions; specifically EEG datasets suffer from low spatial resolution but have a high temporal resolution, whereas fMRI datasets have a high spatial resolution that provides a deeper look into the subcortical structures of the brain but is limited by low temporal resolution. The relative strengths and weaknesses of these two neuroimaging modalities make their combination complimentary for brain analyses.

As for the combination of different modalities, recent studies on tasks different to inner-speech decoding have shown a possible improvement of the neural decoding performance^41–44^. Perronnet^41^ found that haemodynamic and electrophysiological activity during motor imagery tasks was higher when combining EEG and fMRI data compared to when EEG or fMRI data were used alone. Lioi’s^43^ neurofeedback-based dataset of bimodal motor imagery was acquired with simultaneous EEG-fMRI recordings; the data were recorded from 30 subjects performing kinaesthetic motor-imagery tasks with the right hand to bring a ball to a target. In this work, the simultaneous bimodal EEG and fMRI dataset shows the potential of improving the quality of neurofeedback during a motor-imagery task compared to when using only one modality. Berezutskaya^44^ created a publicly-available multimodal nonsimultaneous dataset consisting of ECoG and fMRI data involving naturalistic simulation with a short audio-visual film; the dataset contains ECoG data from 51 subjects (5–55 years of age) and fMRI data from 30 participants (7–47 years of age) on the same task, enabling between-modality and subject-similarity analyses. This bimodal dataset shows the potential of combining different modalities to improve the study of neural mechanisms during language understanding and perception. The major outcomes of these studies were an improvement of the analysis when data from different modalities were combined. Making use of modality combination, hence, would be promising for inner-speech decoding as well.

The closest related work to this study is Cooney^42^, which generated a bimodal dataset of EEG (64-channel) and fNIRS (8-channel) data by acquiring simultaneous recordings from 19 subjects during outer and inner speech. However, the improvement in the performance in the task of inner-speech decoding was not as significant. Specifically, fNIRS showed a low decoding performance, and the use of the fNIRS modality was proven not significant for the bimodal decoding. Therefore, the choice of this work is to focus on EEG without fNIRS.

In terms of simultaneous vs nonsimultaneous recordings, we decided to choose nonsimultaneous recordings. Following the assessment procedure proposed by Scrivener^45^, we weighed in the following reasons: the analysis does not require simultaneously recorded data and it is not acceptable that the EEG data contain more artifacts when recorded with fMRI. Furthermore, nonsimultaneous recordings enable optimization of the task for each modality, such as fast paradigms with EEG and slow paradigms with fMRI, which has a slow BOLD response, therefore optimal for the bimodal acquisition of EEG and fMRI when comes to the inner-speech task.

The aim of this study was to collect separately-recorded EEG and fMRI recordings from healthy participants, performing an inner-speech task that followed the same experimental protocol for both modalities. This study showed that combining separately-recorded EEG and fMRI data can facilitate the decoding of inner speech, as this approach combines both high temporal and spatial resolution. To the best of our knowledge, this study represents the first publicly available dataset with bimodal nonsimultaneous EEG and fMRI recordings of inner speech. This bimodal dataset will allow future users to investigate the potential advantages of using bimodal *versus* unimodal data for inner-speech recognition and will also contribute towards the BCI development in the area of speech prostheses.

## Methods

### Participants

In order to identify participants for our study, we announced the study by distributing flyers describing the experimental procedure and aim of the study, at the Lulea University of Technology, following a list of predefined inclusion and exclusion criteria. In particular, the following inclusion criteria were followed: The current study aimed for an even gender distribution. In order to homogenize the sample, only right-handed people were considered in the study. To facilitate communication during data collection, which is mainly carried out by a non-Swedish-speaking person, primarily English speakers were consulted. In the same manner, the following exclusion criteria were followed: If people have difficulty understanding or following the instructions given at the time of preparation or if for some reason they were feeling uncomfortable during the magnetic resonance imaging (MRI) or EEG examination, they were excluded from the study. All participants filled out an fMRI pre-screening form in order to exclude people that should not undergo the experimental procedure (due to the presence of metallic objects in their body, claustrophobia etc.). The study was approved by the *Swedish Ethical Review Authority (Etikprövning myndigheden, ID:2021-06710-01)* (https://etikprovningsmyndigheten.se/) in accordance with the Swedish Ethical Review Act (SFS 2003:460). Ten participants filled out the questionnaire and as our ethical approval allowed only for a limited amount of subjects, we decided to include only right-handed subjects covering both genders (at least 40% of each gender). As a result, five healthy right-handed subjects aged 33–51 years, participated in this study (three females and two males). Detailed information on the subjects in this study is shown in Table 1. None of these subjects were native English speakers.

**Table 1:** Participant characteristics.

Participant	Self-declared sex	Age	Handedness	Native language
sub-01	Male	33	Right	Hindi
sub-02	Male	35	Right	Bengali
sub-03	Female	51	Right	Greek
sub-04	Female	35	Right	Arabic
sub-05	Female	37	Right	Hindi

All subjects followed the same experimental protocol for the two modalities. The acquisition of the EEG and fMRI recording were performed sequentially followed the general approach of having an EEG recording followed by a fMRI recording with at least one hour of relaxation in between. In this study, we refer to the subjects with the following naming convention: sub-01, sub-02, sub-03, sub-04 and sub-05. Due to high fluctuations during the EEG recording the data from sub-04 were excluded from this study. All subjects provided written consent to participate in the study and to publish this dataset.

### fMRI hardware and setup

The data were collected using a Siemens Magnetom Prisma MRI system (Siemens Healthineers, Erlangen, Germany), equipped with a 20-channel head coil. The visual stimuli were presented during the fMRI recording from a computer to an Ultra HD LCD display (NordicNeuroLab, Bergen, Norway). The screen was 88 × 48 cm (3,840 × 2,160 pixels at full resolution).

Anatomical images were acquired using a sagittal T1-weighted 3D magnetization-prepared rapid acquisition gradient echo (MPRAGE) sequence with the following parameters: repetition time (TR) = 2300 ms; echo time (TE) = 2.98 ms; inversion time (TI) = 900 ms; flip angle = 9; slices = 208; matrix size = 256 × 256; and voxel size = 1 × 1 × 1 mm. Right after the anatomical scan, two field maps were obtained (A and B) with the following parameters: TR = 662.0 ms, TE: A = 4.92 ms, B = 7.38 ms; and voxel size = 3 × 3 × 2 mm. Next, functional MRI was obtained using an echo-planar imaging sequence with a multiband acceleration factor = 2 and in-plane acceleration factor = 2, parallel to the bicommissural plane with the following parameters: TR = 2.16 s; TE = 30 ms; slices = 68; matrix size = 100 × 100 and voxel size = 2 × 2 × 2 mm.

### EEG hardware and setup

The EEG data were acquired using the *BioSemi Active2* measuring system (BioSemi B.V., Amsterdam, Netherlands) with a 16-bit resolution and a sampling rate of 512 Hz. A BioSemi EEG head cap with 64 electrodes in pre-fixed electrode positions and 6 external sensors was used. An appropriate cap size was selected for each participant by measuring his or her head circumference from nasion to inion. We also ensured that the cap was properly centred with the *C*_*z*_ (Vertex) at the centre of the head, namely, halfway between the nasion and inion and halfway between the two ears. *SignaGel* (Parker Laboratories BV, Almelo, Netherlands) was applied to each electrode to provide electrode connectivity with the subject’s head. All six external electrodes (EXG1-EXG6) were placed using stickers. The locations of the six electrodes were as follows:

- EXG1: On the left mastoid behind the left ear
- EXG2: On the right mastoid behind the right ear
- EXG3: 1 cm to the left of the left eye (aligned to the centre of the eye)
- EXG4: 2 cm above the left eye (aligned to the centre of the eye)
- EXG5: 2 cm below the right eye (aligned to the centre of the eye)
- EXG6: 1 cm to the right of the right eye (aligned to the centre of the eye)

EEG data were recorded with ActiView software, which was also developed by BioSemi. ActiView enables verification of the electrode impedance as well as the overall quality of the incoming data. The impedance of each electrode was manually examined at the beginning of each recording session, to ensure that it was between −20 µV and 20 µV; any electrodes not within this range were adjusted before recording to ensure the correct impedance, by adding/removing some gel, moving the participant’s hair underneath the electrode or wiggling the electrode. Lights in the room were dimmed to avoid subject’s eye flickering due to the high contrast between the room and the visual display.

### Experimental protocol

**Fig. 1:**
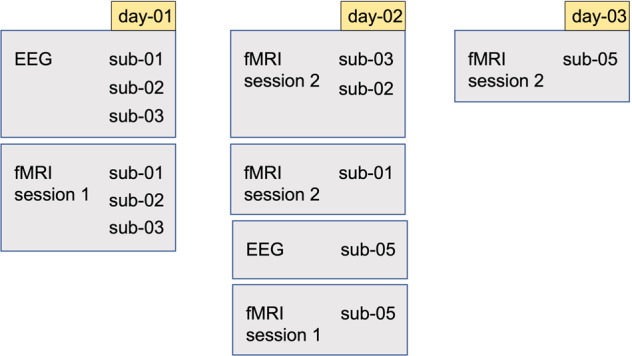
EEG /fMRI schedule - The overall experimental protocol performed over a period of 3 consecutive days. Day-01 contains the EEG recordings as well as the recordings of the first fMRI session of sub-01, sub-02, and sub-03. Day-02 contains the fMRI recordings for session 2 of sub-01, sub-02, and sub-03, the fMRI recordings for session 1 of sub-05 and the eeg recordings of sub-05. Day-03 contains the fMRI recordings for session 2 of sub-05. There was always a relaxation period of at least one hour in between the recordings and proper time for a break to avoid participants’ fatigue.

The overall experimental protocol consisted of two fMRI sessions and one EEG session and was performed over a period of 3 consecutive days. In this study, all EEG recordings were performed first, as the EEG setup can sometimes induce difficulties (achieving good electrode connectivity with the participant’s head). During day-01, the EEG recordings of sub-01, sub-02 and sub-03 took place followed by the recordings of the first fMRI session. The majority of the fMRI recordings for the second session were performed during day-02; only the recordings of sub-05 were performed on day-03. There was always a relaxation period of at least one hour in between the recordings and proper time for a break to avoid participants’ fatigue. The detailed EEG/fMRI schedule is illustrated in Fig. 1.

**Fig. 2:**
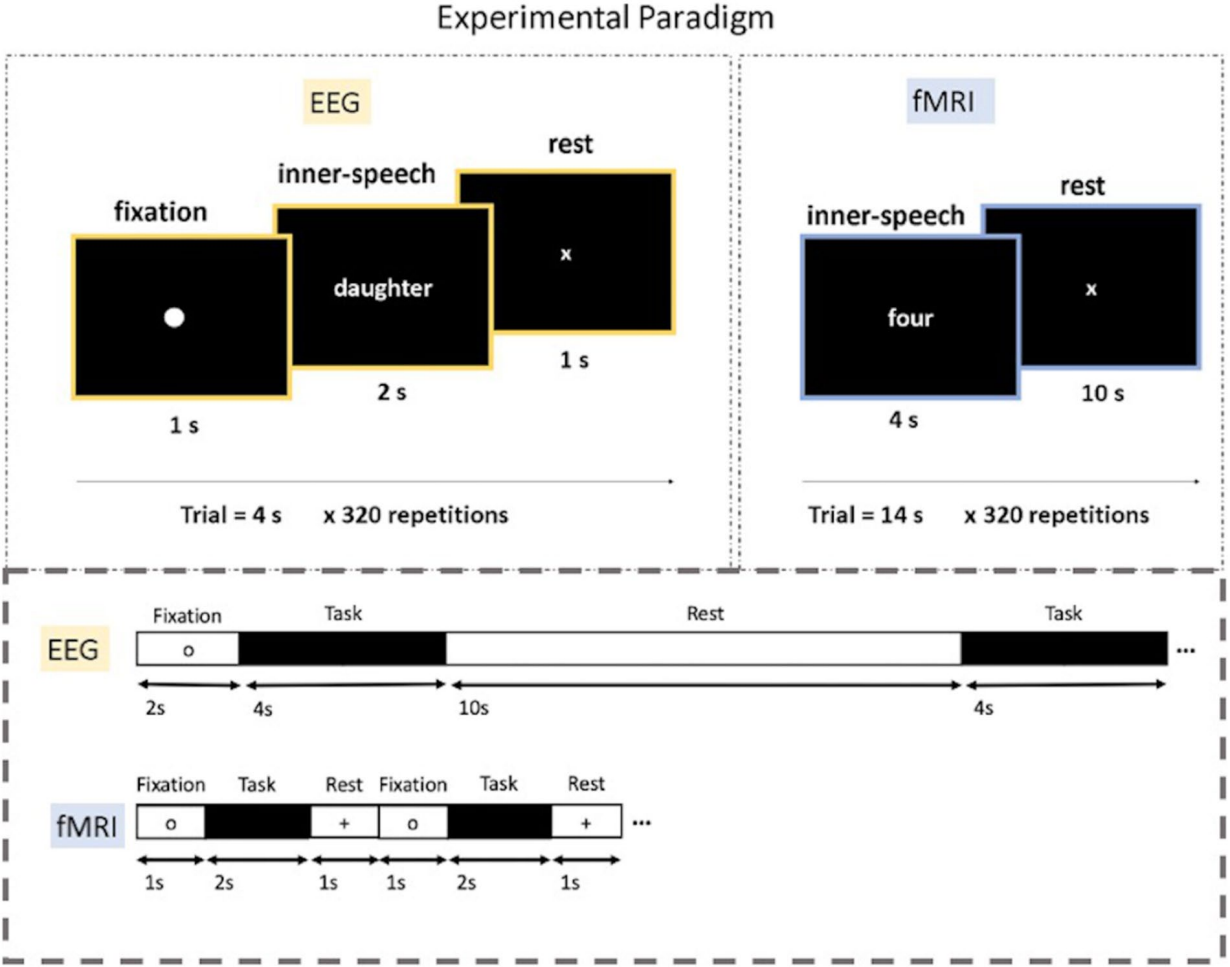
Experimental paradigm - The trial designs are depicted in the top part of the figure and the respected timelines for the trials are shown at the bottom. During the fMRI inner-speech task, participants were requested to think of the presented word as many times as possible. There were 320 trials in total, split into two sessions of 160 trials each. For the eeg recordings, there was only one session, and the participants were instructed to use their inner-speech only once during the inner-speech task interval. Note that the rest period for the fMRI protocol was longer than that for the eeg protocol.

The experimental protocol for both modalities (fMRI and EEG) was designed using E-Prime 3.0^46^ and is illustrated in Fig. 2. Huth^21^ shows that semantically selective brain areas appear to be organised in the same manner across individuals and provides word frequency statistics for the text corpus employed. Based on the reference study, two categories, social and number, with four words each were selected. The two selected categories were mapped into different brain areas and the selected words appear to have a high word co-occurrence frequency. The social category contained the words *child, daughter, father, and wife*. The number category contained the words *four, three, ten, and six*. The textual representation of the words was presented randomly on the screen in front of the participant. There were a total of 2,080–2,200 fMRI volumes collected per subject, divided into two sessions. Each volume contained 100 × 100 × 68 voxels. The EEG recordings provided a total of 320 × 64 × 1,024 samples per subject.

#### fMRI procedure

The fMRI recordings consisted of two sessions performed over a period of three days. At the beginning of each session, written instructions for the experiment were presented on the screen until the participants informed the fMRI operator through an intercom that they are ready to proceed with the experiment. A fixation period of 2 s was followed, in which the participants were instructed to fixate their eyes on the centre of the screen. The 2 s fixation time was included only once at the beginning of each session since there was a significant rest period of 10 seconds between trials. Then, each trial consisted of the inner-speech task (4 s) and a subsequent rest period (10 s). Eight different words were used for the inner-speech task, divided into 2 categories (social or number words), and there were 20 trials for each word in each session; thus, each session consisted of 160 trials. During the inner-speech task, the word stimulus was presented in white font against a black background for 4 s, and the participants were encouraged to repeat the given word in their minds as many times as possible (approximately 4 times) without any accompanying articulation or muscle movement (i.e., using their inner speech). The word stimuli were presented in a randomized order over the 160 trials. During the rest period, a white fixation cross was presented for 10 s, and the participants were allowed to relax and prepare for the next trial. The total duration of the recordings for the 320 repetitions was 74.6 min per participant.

#### EEG procedure

The EEG recordings consisted of one session with 40 trials per word, using the same stimuli as in the fMRI protocol, that was performed before the fMRI acquisition for all subjects but for sub-05 due to time constraints of the recording facility. During the experiment, the participants were seated in a comfortable chair in front of a computer screen where the experiment was visualized. The procedure took place in a dark, electrically shielded room, with the operators seated outside the room. To familiarize the participant with the experimental procedure and the room environment, all steps of the experiment were explained at the start of the experiment. The participants were instructed to use only their inner speech, to not move their eyes or head while the stimuli were presented, and to try to stay still during the experiment. Participants were given the option to pause the experiment at any time if needed. The EEG operators were able to visually monitor the participant through a dark window for any signs of fatigue, but the participant was unable to see the operators. Additionally, the operators continuously monitored the quality of the EEG signals during recording to detect any major artifacts caused by eye or motion movements. At the beginning of the session, the written instructions for the experiment were presented on a screen to the subject until they pressed the spacebar to start the experiment. Each trial included fixation, task, and rest periods, with durations of 1 s, 2 s, and 1 s, respectively. During the fixation period, the participant was instructed to direct their gaze to the centre of the screen, where a small circular fixation point was located. During the task period, the word stimulus was presented for 2 s, and the participants were asked to repeat the stimulus in their minds without any accompanying articulation or muscle movement (i.e., using their inner speech). During the rest period, the participants were allowed to relax and prepare for the next trial. The total duration of the recording, which contained 320 repetitions, was 21.33 min per participant. Note that the rest period for the fMRI protocol was longer than that for the EEG protocol because the fMRI BOLD signal typically peaks approximately 5 s after stimulus onset and takes approximately 14 s to recover to baseline levels^47^.

### fMRI Preprocessing

The fMRI data were preprocessed with SPM12^48^. First, spatial displacement maps were calculated for each session. These were used for motion correction of the functional data. Slice-timing correction was performed as the fMRI data were acquired in an interleaved order. Next, images were coregistered to the T1-weighted structural scan with a normalized mutual information cost function. Prior to normalization, these images were used for within-subject classification.

To verify that neural activity related to inner speech and the two semantic categories (social and number words) was as expected, further processing was conducted (see “fMRI activation – group level”). The origin was manually set to the anterior commissure, followed by normalization to the Montreal Neurological Institute (MNI) space. Smoothing using an 8-mm full-width at half-maximum (FWHM) Gaussian kernel was applied. We estimated a general linear model (GLM) convolved with a canonical haemodynamic response function. The category regressors (social or number word) were time-locked to the onset of its respective inner-speech word with a duration of 4 s. The rest of the regressors had durations of 10 s. Nuisance variables, such as movement parameters calculated in the previous realignment step, were also included. This GLM enabled investigation of the activation at the subject level; subsequently, a fixed effect analysis was applied to determine activation at the group level. The planned comparisons included inner speech and rest (inner speech – rest) and the stimulus category (number word – social word; social word – number word). For all analyses, the extent cluster threshold was ^*K*^*E* > 20 with a familywise error (FWE) correction of *p* < *0.05* at the voxel level.

### EEG preprocessing

In the current work, we utilized EEGLAB^49^ to preprocess the EEG data subject-wise. EEGLAB is a MATLAB toolbox for processing continuous and event-related EEG, MEG, and other electrophysiological data. The toolbox has features such as independent component analysis (ICA), time/frequency analysis, artefact rejection, event-related statistics, and several useful visualization modes for averaged and single-trial data.

The raw BioSemi EEG data in .bdf format were imported to EEGLAB using reference channel 48 (Cz). A multitude of internal and environmental causes can generate temporal drifts, which change over time and across the electrodes. To reduce the impact of such variances, it is usual practice to perform a so-called baseline correction. In this study, baseline correction was applied using a zero-phase finite impulse response (FIR) high-pass filter at 1 Hz. Low-frequency and high-frequency signals, which are commonly caused by environmental/muscle noise in scalp EEG and are not usually the focus of analysis, were filtered out.

Noise below and above a given frequency was retained using low-pass and high-pass filtering. Here, we applied zero-phase finite impulse response (FIR) bandpass filtering with 1 Hz (lower edge) and 50 Hz (higher edge) boundaries of the frequency bandpass, eliminating the requirement for a notch filter. Rereferencing facilitates data cleaning by providing an estimate of physiological noise at baseline. In this study, rereferencing was performed using the average reference to Cz, excluding channels 65–70 (the mastoid and ocular electrodes); averaging referencing was chosen over rereferencing from the mastoid electrodes to guard against introducing any signal artifacts which may have resulted from differences in placement of the external electrodes between participants (as per, for example, our decision to disregard the data from subject 4 (sub-04), to ensure overall data integrity).

**Fig. 3:**
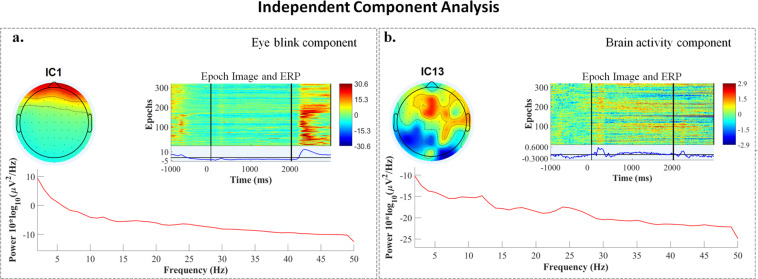
Components decomposed by ICA, showing eye blinks and brain activity for subject 5, before artifacts removal. Top left: topography map illustrating the projection of the independent component activity. Center: event-related potentials of the independent component and its associated power, per epoch. The times marked with vertical lines indicate the start of the fixation, inner-speech task, and rest periods (at −1,000 ms, 0 ms and 2,000 ms respectively). Bottom: spectrum of the independent component.

**Fig. 4:**
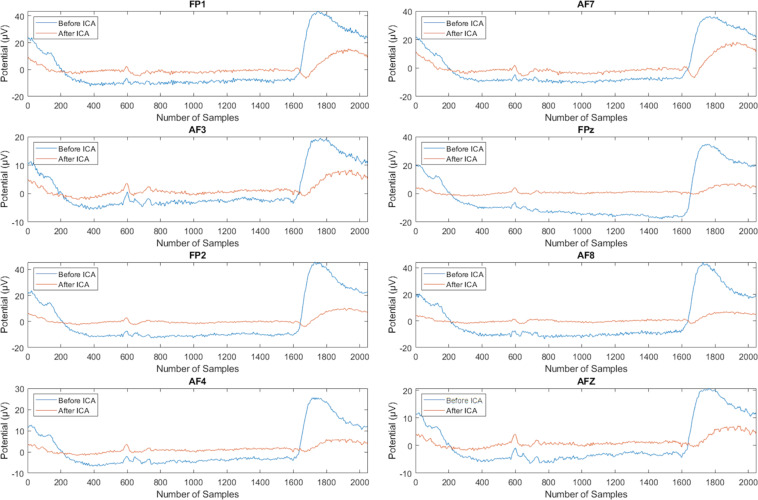
Illustration of eye artifacts reduction in the frontal region. EEG signal before (blue) and after (red) applying ICA.

Channels were manually inspected, and bad channels were rejected and not interpolated. Time-locked epochs were extracted using start-stop limits fixed within the interval [0, 2 s]. ICA can be used to identify data segments strongly influenced by motor-related artifacts, such as eye blinking and movement of the jaw, neck, arm, or upper back, for removal. Figure 3 illustrates two out of the 64 ICA components before artifact removal. The top-left is the ICA component showing blink/brain activity; the center part shows the activity with respect to all 320 trials, and the blue graph shows the aggregated activity across all trials (ERP). The red graph in the bottom part is the power spectrum. The effect of the eye artifact removal in the frontal region after applying ICA is shown in Fig. 4. It can be noticed that eye artifacts have been significantly reduced after ICA. In this study, we examined the topography as well as the spectrogram and frequency variation to decide whether a component should be retained. In examining the topographies, high activity in the far-frontal projections is a strong indicator of electrooculography (EOG) artifacts; in the spectra, decreasing power with a slope that is more shallow and spread more evenly over the frequency range is also a strong indicator of an EOG artifact: taken together (along with the ocular reference channels, 67–70, which serve to provide the EOG signal pattern that the independent components are matched against) we reliably identified artifact-related independent components to zero-out from the data, as part of a manual inspection and cleaning process that is often more reliable than algorithmic methods relying on peak-to-peak signal information, which may vary greatly between subjects. In this study, the preprocessing has been performed in a subject-wise manner.

Finally, we extracted epochs according to the *time-locking event*. We performed this process manually, with the epoch limit [0,2 s], this interval encapsulated the main activity.

## Data Records

The raw anonymized EEG and fMRI data of the four subjects are available in *Brain Imaging Data Structure* (BIDS) format^50,51^ (https://bids-specification.readthedocs.io/en/stable/) at the OpenNeuro repository^52^. The data for each subject are organized into three sessions: two for the fMRI modality (ses-01 and ses-02) and one for the EEG modality (ses-EEG). Each of the 8-word stimuli were assessed with 40 trials, resulting in 320 trials in each modality for each subject. There are 4 directories, one for each subject (sub-01, sub-02, sub-03, and sub-05). The data from sub-04 were deemed unfit for use and thus were not made available.

### fMRI

All Digital Imaging and Communications in Medicine (DICOM) files with fMRI data were converted into Neuroimaging Informatics Technology Initiative (nifti) format using *MRIcroGL v1.2.0211006* (https://www.nitrc.org/plugins/mwiki/index.php/mricrogl) and then organized into BIDS format. Each .nifti file is accompanied by the corresponding .json file. The anatomical image consists of the anatomical scan with file names in the following format: sub-*XX*_*YY*-T1w, where *XX* denotes the subject ID and *YY* denotes the session ID. The fmap folder consists of three .nifti files: two files are for magnitude, and one is for phase difference. The two magnitude files are named with the following format: sub-*XX*_*YY*_magnitudeZ, where *XX* denotes the subject ID, *YY* denotes the session ID, and Z takes the value 1 for *TE*1 = 4.92 *ms* and 2 for *TE*2 = 7.38 *ms*. The .nifti file for the phase difference image is named with the following format: sub-*XX*_ses-*YY*-phasediff, where *XX* denotes the subject ID and *YY* denotes the session ID. The functional data are available in the func folder, where the .nifti file and its corresponding .json file have the following format: sub-*XX*_ses-*YY*_task-inner-bold, where *XX* denotes the subject ID and *YY* denotes the session ID. The task event file is also available as a .tsv file named sub-*XX*_ses-*YY*_task-inner-events, where *XX* denotes the subject ID and *YY* denotes the session ID in the corresponding func folders.

### EEG

The raw EEG data collected in one session are available in .bdf format in the ses-EEG folder for each subject. The .bdf file was exported using EEGLAB software v2021.1, with sampling rate of 512. Each event, the corresponding ID and description are presented in Table 2. The task events are provided in a .tsv file for each subject in the respective ses-EEG folder.

**Table 2:** Event IDs and their accompanying descriptions.

EventID	Description
1	fixation
2	rest
111	child
112	daughter
113	father
114	wife
125	four
126	three
127	ten
128	six

## Technical Validation

### EEG

**Fig. 5:**
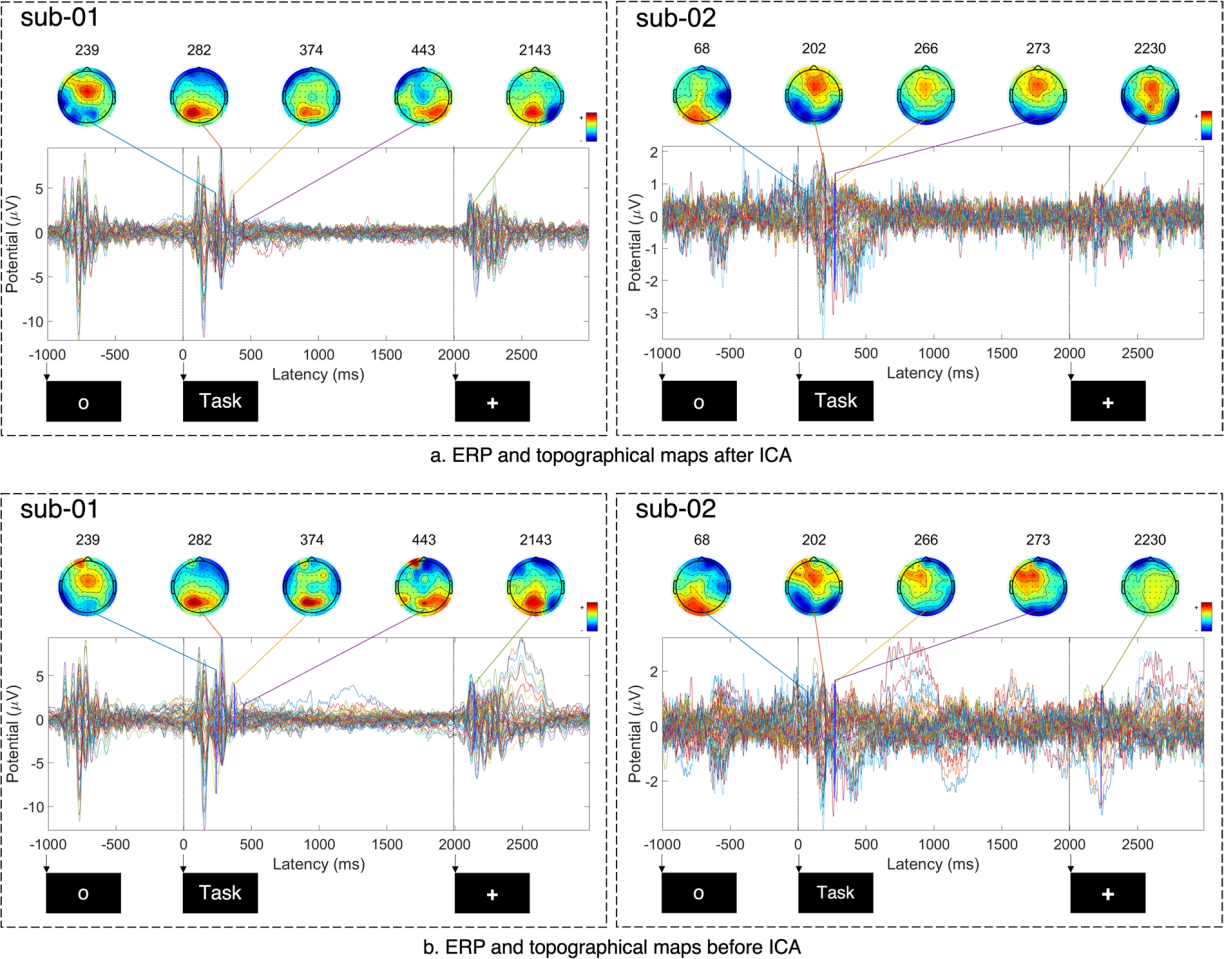
Plots of event-related potentials and topographical maps of activity before (b) and after (a) artifact removal using ICA for subjects 1 (sub-01) and 2 (sub-02). All plots are created from preprocessed data by averaging over 320 trials for each subject. The 64 coloured waves correspond to the 64 EEG channels. The time axis shows the duration of a single trial and corresponds to a total duration of 4,000 ms. Notably, the time axis starts from a negative value of t = −1,000 ms, which corresponds to the 1,000 ms fixation period at the beginning of a trial. The times marked with arrows indicate the start of the fixation, inner-speech task, and rest periods (at −1,000 ms, 0 ms, and 2,000 ms, respectively).

**Fig. 6:**
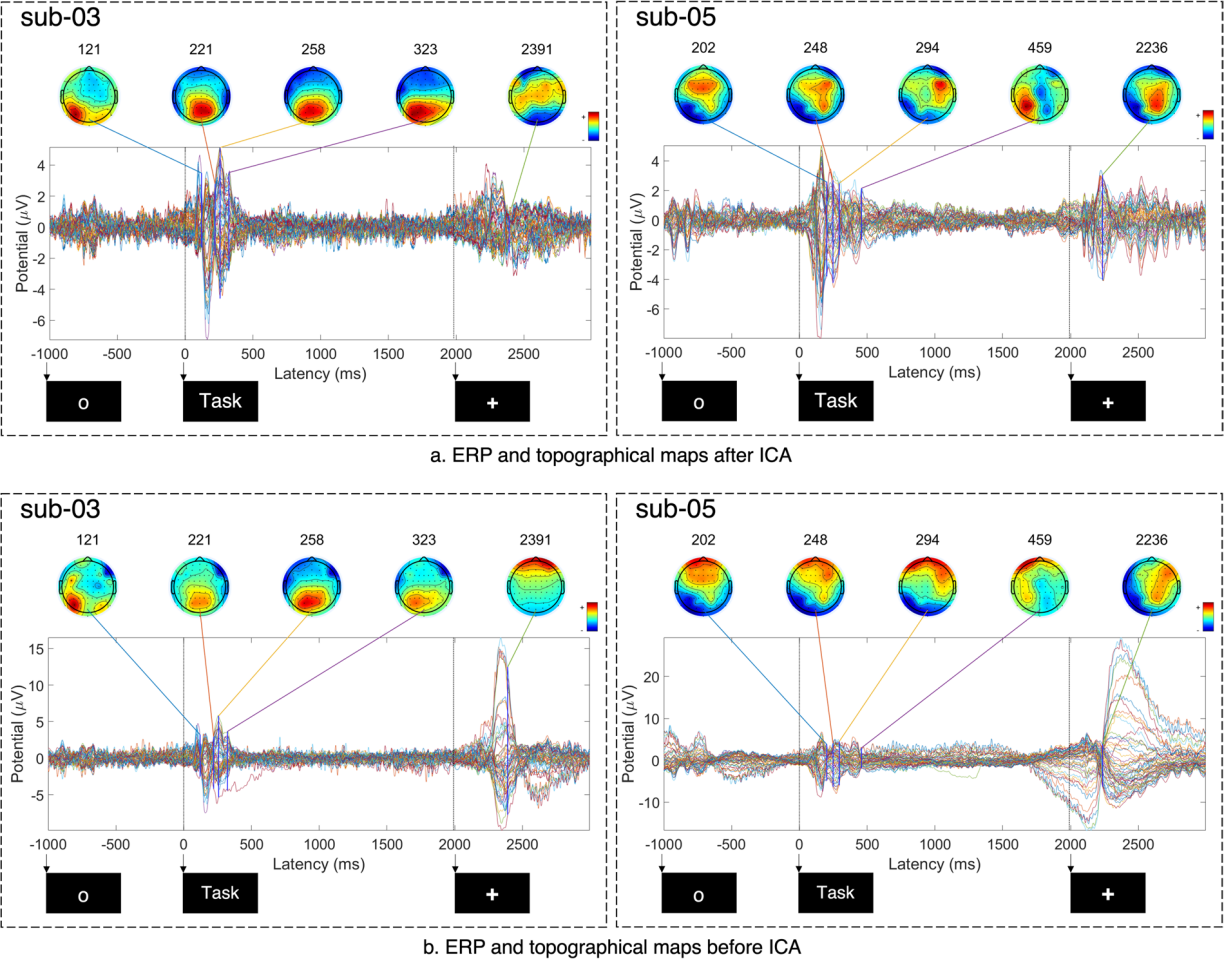
Plots of event-related potentials and topographical maps of activity before (**b**) and after (**a**) artifact removal using ICA for subjects 3 (sub-03) and 5 (sub-05). All plots are created from preprocessed data by averaging over 320 trials for each subject. The 64 coloured waves correspond to the 64 EEG channels. The time axis shows the duration of a single trial and corresponds to a total duration of 4,000 ms. Notably, the time axis starts from a negative value of t = −1,000 ms, which corresponds to the 1,000 ms fixation period at the beginning of a trial. The times marked with arrows indicate the start of the fixation, inner-speech task, and rest periods (at −1,000 ms, 0 ms, and 2,000 ms, respectively).

An event related potential (ERP) is the measured brain activity in response to a stimulus, therefore suitable for verifying the technical validity of the data. Figures 5, 6 shows the ERP activity for each subject before and after applying ICA subject-wise.

From the subplots after ICA, it can be observed that more pronounced potential deflections occur after stimulus presentation (after t = 0 ms); however, a small range of deflection can also be noticed within the two other periods (rest and fixation); the presentation of new visual stimuli to the subjects, to signal the start of each period within each trail, has resulted in these evoked responses (so-called fixation-onset ERPs^53^).

The activity at 239 ms, 282 ms, 374 ms, and 443 ms for sub-01; at 68 ms, 202 ms, 266 ms, and 273 ms for sub-02; at 121 ms, 221 ms, 258 ms, and 323 ms for sub-03; and at 202 ms, 248 ms, 294 ms and 459 ms for sub-05 indicate prominent brain activity stimulus onset. Data from both subjects followed similar pattern during the inner-speech task (0–500 ms). As shown in the Figs. 5, 6, strong positive and negative deflections occurred during 0–500 ms, which indicates the evoked response upon presentation of the visual stimulus, and increased brain activity during the performance of the inner-speech task. These evoked responses indicate strong P300 and N400 components, which are also observed in similar trials in the related research area of imagined speech^54^.

**Fig. 7:**
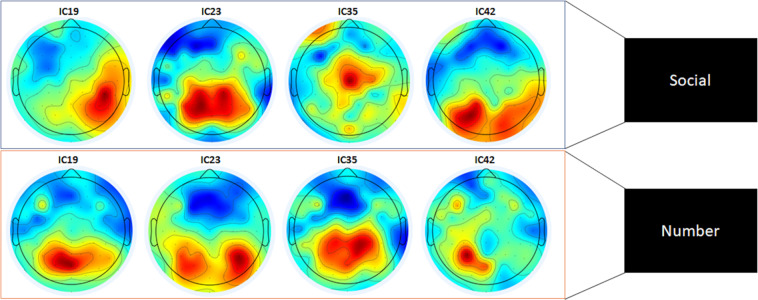
Topological maps for subject 1 (sub-01) corresponding to stimuli from the number and social categories for components 19, 23, 35, and 42.

**Fig. 8:**
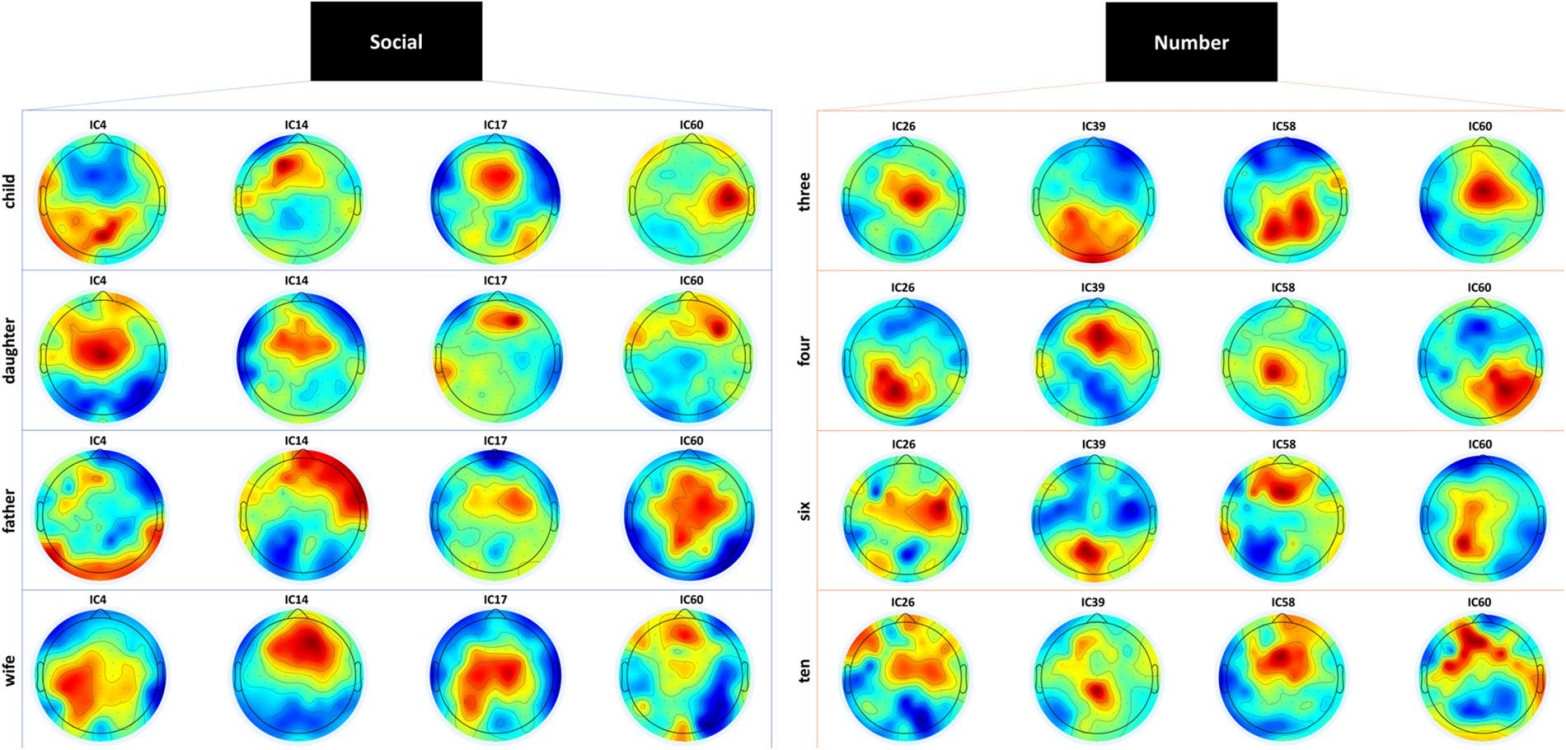
Topological maps for subject 1 (sub-01) for each stimulus in the number and social categories.

Next, to provide a more detailed analysis of the activated brain areas, we generated topological maps (after ica decomposition). Figure 7 depicts the activation in response to stimuli in the two semantic categories (number or social words) for all subjects in topological maps. As shown in the figure, it appears that these activities were mainly dominated in central regions. Notably, the activity in response to all stimuli (eight words) for sub-01 is shown in the topological maps in Fig. 8. In this figure, 4 ICs with high brain activity are shown for all words. For a specific IC, regions were differently activated for each of the eight words. The topographic projections of each word illustrate the average difference in brain activity between the inner-speech task of each word; the variance between these projections confirms the subject’s different activation regions, both between and within our semantic categories, which further validates the data.

Finally, a comparison of the ERPs and topological maps of brain activity before and after artifact removal using ICA subject-wise is shown in Figs. 5, 6. From the plots it is noticable that the eye artifacts have been significantly reduced after applying ICA compared to before applying ICA. Furthermore, the Pearson product-moment correlation coefficient^55^ between the EEG and EOG channels before and after artifact removal is reported in Table 3. The values indicate that EOG signals have a relatively higher correlation with the EEG signals before artifact removal (column 2) rather than after artifact removal with ICA (column 3), especially for subjects sub-01, sub-03, and sub-05. As the typical use of the dataset is subject-dependent analysis, we leave it up to the users to either include sub-02 or not (as sub-02 has the strongest impact of eye movement/blinking).

**Table 3:** Pearson product-moment correlation coefficient between the EEG and EOG channels with and without applying ICA.

Subject	Before artifact removal	After artifact removal (ICA)
sub-01	0.44	0.29
sub-02	0.93	0.87
sub-03	0.68	0.33
sub-05	0.92	0.60

### fMRI activation - group level

**Fig. 9:**
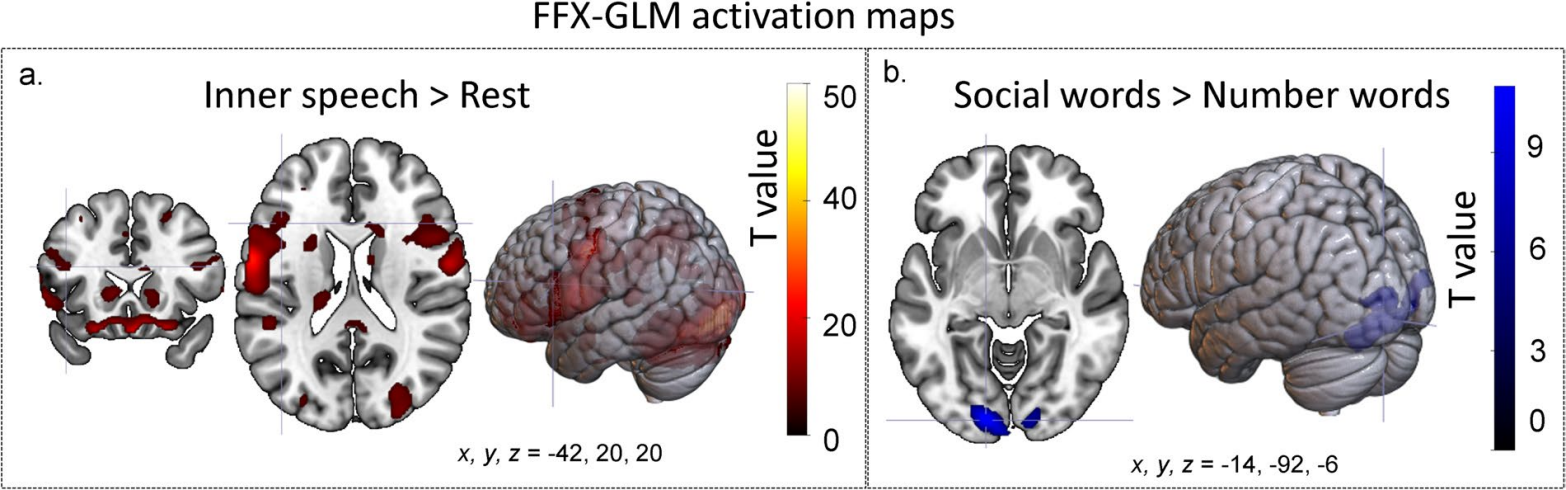
Activation maps generated from a group-level fixed-effects model for the following comparisons: (**a**) areas more highly activated during the inner-speech task than the resting condition (number and social words combined) – this slice is selected to highlight activation in Broca’s area (coordinates: −42, 20, 20), and (**b**) areas more highly activated by social words than number words – this slice is selected to highlight activation in the secondary visual cortex (coordinates: 14, −92, −6). An increase in activation was found for the reverse contrast (i.e., increased activation for number words over social words).

In this study, group-level analyses were conducted to verify that the inner-speech task activated neural regions connected to inner speech. As expected, the inner-speech task activated language- and orthographic-related regions when compared with the baseline rest condition. The increased activity during inner speech (see Fig. 9a and Table 4) is indicated by average BOLD activation displaying significant activation in areas directly related to language processing, including Wernicke’s area (Brodmann’s area (BA) 22) in the left hemisphere and Broca’s area and surrounding regions. Further activation was found in the left supramarginal gyrus, which (alongside the pars opercularis) has been implicated in inner speech^56^, and the angular gyrus, which is related to semantic processing^57,58^. Areas of high activation also included visual processing regions, such as the bilateral secondary visual cortex and the right frontal eye fields, and orbitofrontal area. These regions likely relate to processing of visual word forms, as the word cue was presented orthographically. Not surprisingly, motor regions, including the primary motor cortex and premotor and supplementary motor cortices, were activated by the inner-speech task. Motor activity still occurs during inner-speech^59^, albeit at reduced activation levels compared to outer speech (spoken aloud)^60^. These findings support the reliability of the data as they indicate that the inner-speech task activated language, orthographic, and motor related regions.

**Table 4:** Areas with increased activation during inner speech relative to baseline (the rest condition).

Anatomical area	BA	Hemi	(x y z)	^*K*^*E*	Peak T Value	^*z*^*E*
Secondary visual cortex	18	L	−22, −96, −6	10470	54.5	Inf
	—	R	22, −98, 2	—	41.4	Inf
	—	R	30, −92, 2	—	38.89	Inf
Primary motor cortex	4	L	−50, −12, 44	1595	23.16	Inf
	—	L	50, −6, 42	—	16.57	Inf
	—	L	−56, −4, 20	—	14.77	Inf
Premotor cortex/supplementary motor area	6	R	50, −6, 42	1515	20.59	Inf
	6	R	50, −6, 56	—	15.37	Inf
Primary motor cortex	4	R	58, −4, 30	—	9.98	Inf
Praecuneus/superior parietal lobule	7	L	−24, −58, 54	2534	15.54	Inf
Supramarginal gyrus	40	L	−36, −40, 44	—	13.66	Inf
Praecuneus/superior parietal lobule	7	L	−20, −68, 44	—	11.32	Inf
Anterior prefrontal cortex	10	R	28, 64, 8	476	15.43	Inf
	—	R	18, 64, 8	—	15.34	Inf
	—	L	−22, 60, 2	—	14.22	Inf
Premotor cortex/supplementary motor area	6	L	−6, −4, 68	965	13.87	Inf
	—	L	−4, 2, 62	—	12.59	Inf
	—	R	6, −2, 70	—	9.36	Inf
Pars orbitalis	47	R	24, 18, −20	60	10.4	Inf
	—	R	32, 28, −18	—	6.82	6.81
Praecuneus/superior parietal lobule	7	R	30, −46, 44	1394	10.32	Inf
Angular gyrus	39	R	30, −62, 44	—	10.12	Inf
	—	R	28, −52, 52	—	9.8	Inf
Pars orbitalis	47	L	−22, 20, −22	121	9.83	Inf
	—	—	−18, 10, −22	—	7.5	7.48
	—	—	−14, 12, −14	—	5.72	5.72
Pars orbitalis	47	R	44, 30, −14	51	9.67	Inf
Basal ganglia	—	L	−12, 20, 2	1044	9.39	Inf
	—	L	−24, 6, 0	—	7.3	7.3
	—	L	−18, 12, −4	—	7.1	7.3
Premotor cortex/supplementary motor area	6	L	−48, 2, 4	107	8.02	Inf
Superior temporal gyrus	22	L	−52, −40, 16	93	7.88	Inf
Inferior frontal gyrus: pars triangularis	45	L	−48, 22, −2	45	7.46	7.45
Orbitofrontal area	11	R	4, 26, −12	80	6.77	6.76
Primary motor cortex	4	L	−16, −26, 64	65	6.58	6.57
Ventral anterior cingulate	24	R	8, 18, 20	177	6.33	6.33
Inferior frontal gyrus: pars opercularis	44	L	−42, 20, 20	93	6.31	6.3
Global pallidus	—	R	22, −4, 4	31	5.76	5.75
Frontal eye fields	8	R	10, 16, 34	72	5.74	5.73
Pars opercularis	44	R	50, 12, 20	32	5.57	5.56
Thalamus	—	L	−8, −20, 8	35	5.5	5.5

Coordinates are in Montreal Neurological Institute (MNI) space. *BA* = Brodmann’s Area, *Hemi* = hemisphere, ^*K*^*E* = cluster size. The cluster threshold was set to 20, with family-wise error (FWE)-adjusted *p < 0.05*.

Comparison of the two semantic categories (social vs. number words) revealed that social words elicited more activation than number words in the bilateral secondary visual cortex and the right primary visual cortex (see Table 5 and Fig. 9b). No areas showed significantly higher activation for number words compared to social words.

**Table 5:** Areas with increased activation for social words relative to number words during inner speech, i.e. the baseline.

Anatomical area	BA	Hemi	(x y z)	^*k*^*E*	Peak T Value	^*z*^*E*
Secondary visual cortex	18	L	−14, −92, −6	1043	11.91	Inf
	—	—	−20, −98, 8	—	7.37	7.36
	—	—	−36, −90, 10	—	4.75	4.75
Primary visual cortex	17	R	12, −94, −4	321	7.85	7.83
Secondary visual cortex	18	R	26, −96, 12	—	6.08	6.08

Coordinates are in Montreal Neurological Institute (MNI) space. *BA* = Brodmann’s area, *Hemi* = hemisphere, ^*k*^*E* = cluster size. The cluster threshold was set to 20. The family-wise error (FWE) was adjusted to *p < 0.05*.

### fMRI activation - individual subjects

**Fig. 10:**
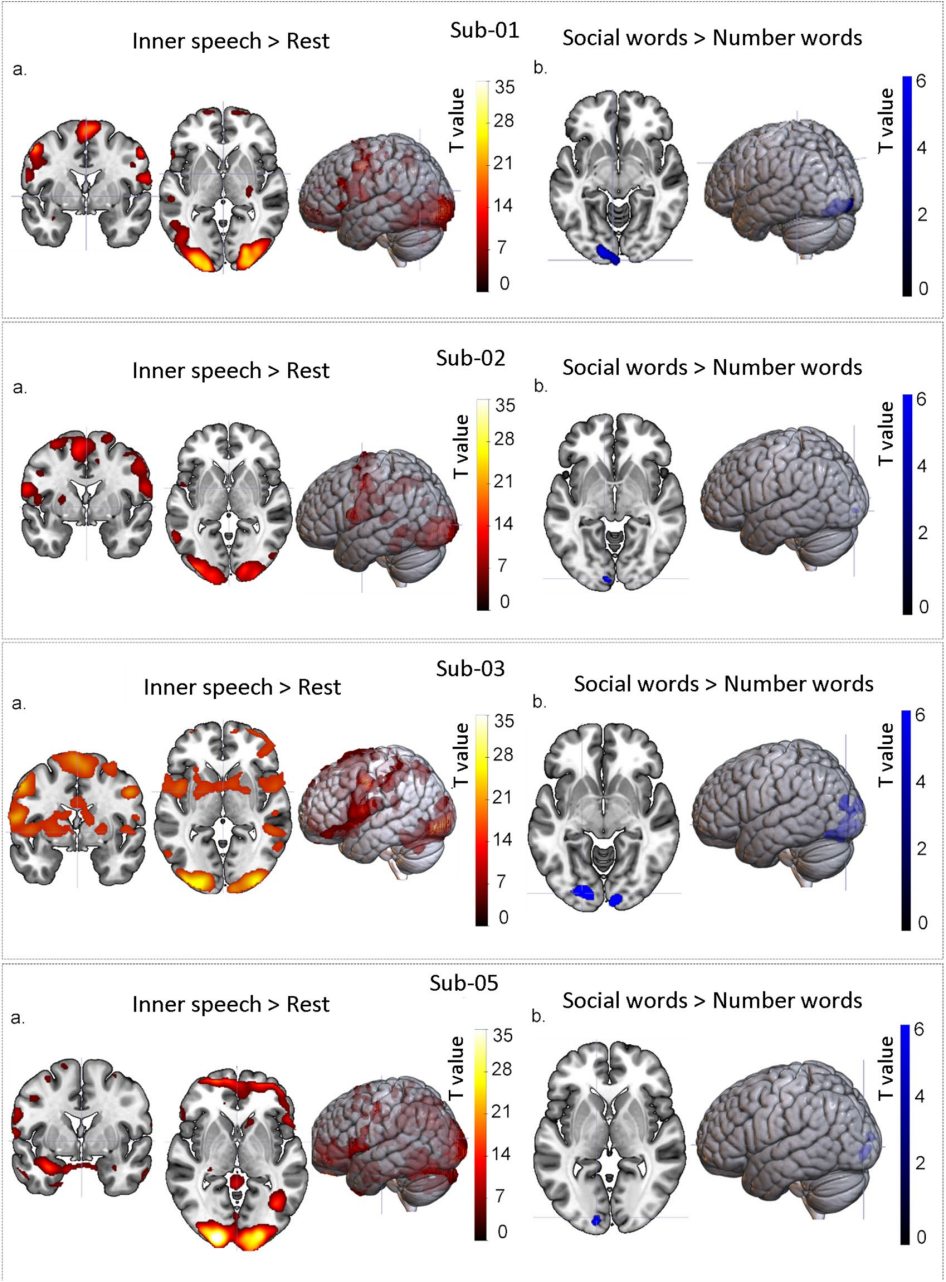
BOLD signals of each subject. (**a**) Area more highly activated during the inner-speech task in the rest condition (numbers and social words combined). (**b**) Areas more highly activated by social words than number words (no areas were more highly activated by number words than social words).

Decoding studies are often conducted within rather than between subjects due to the large extent of individual variance in neural anatomy and functional activity. Therefore, we provided subject-level results to enable researchers to select among subjects based on brain activation profiles. As seen in Fig. 10, the results are consistent across subjects.

### fMRI framewise displacement

**Fig. 11:**
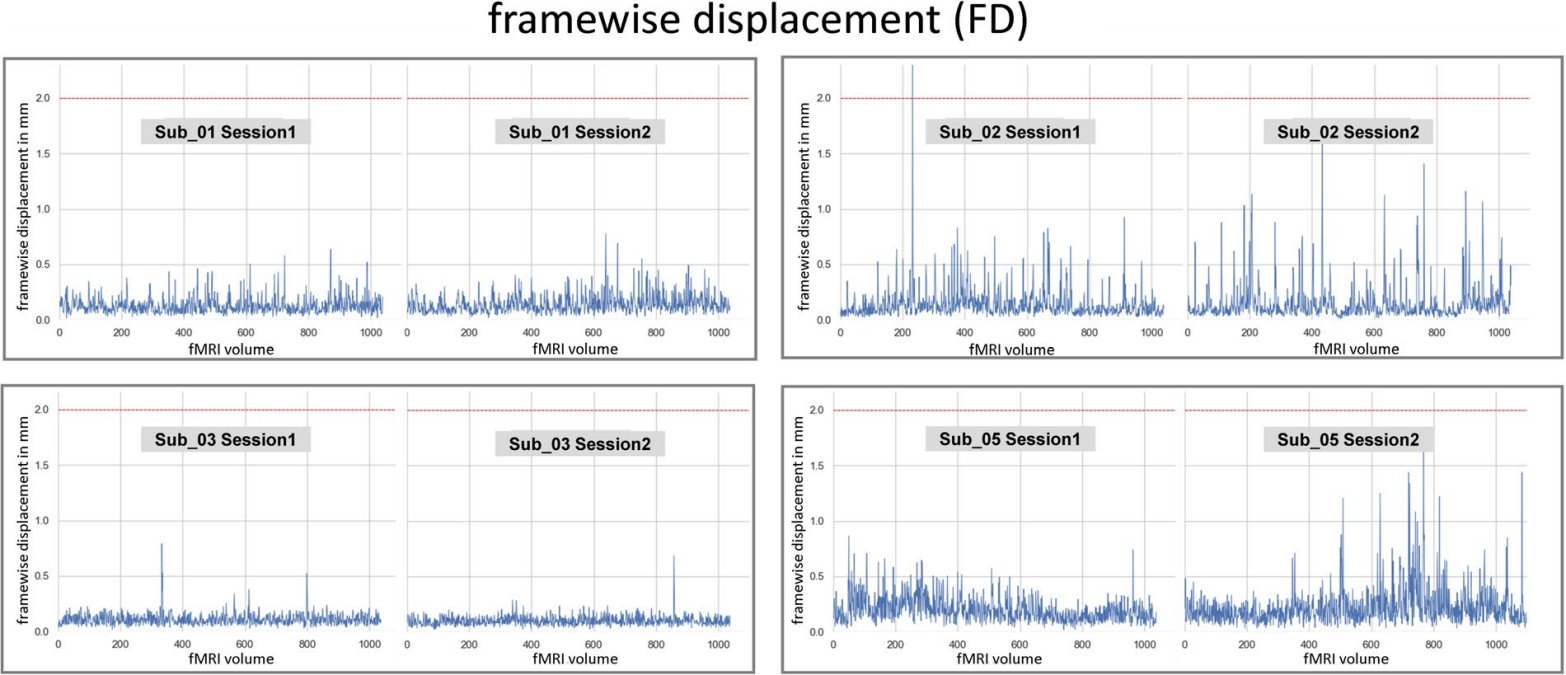
The framewise displacement (FD; in mm) calculated across each subject and session is shown across each volume. The red dashed line indicates the voxel size of the functional images (2 × 2 × 2 mm).

Motion-related artifacts can compromise data quality. Frames that are contaminated with motion above a certain threshold can be rejected by calculating head motion artifacts throughframewise displacement (FD). FD is an overall estimate of movement over time for each subject, which incorporates subtle in-scanner movements. We calculated the FD for each subject and session in Nipype, according to Power^61^. The average FD (in mm) across frames for each subject was as follows: sub-01, session 1 = 0.13 and session 2 = 0.14; sub-02, session 1 = 0.15 and session 2 = 0.14; sub-03, session 1 = 0.11 and session 2 = 0.1; and sub-05, session 1 = 0.2 and session 2 = 0.22; see Fig. 11. There was rarely motion exceeding the size of one voxel (2 × 2 × 2 mm). The analysis also showed that all subjects had a mean fd under 0.25 mm; however, researchers may choose to omit specific volumes with fd values higher than 0.5 mm.

### Limitations

The objective of this study is to provide a unique dataset by merging EEG and fMRI data specifically focused on inner speech. To the best of our knowledge, there is currently no other publicly available dataset that combines EEG and fMRI for inner speech analysis.

There are a couple of limitations to consider in this study. First, the sample size is small, including four participants. Still, datasets with small sample sizes are often a necessary and valuable contribution in novel research paradigms, in which experimental design and decoding strategies are explored and efficacy assessed. For example, other foundational fMRI datasets for neural decoding included just two to four participants^62–65^ and enabled other researchers to use these datasets to greatly advance the field. Second, the participants have different native languages. However, it is important to note that the primary goal of this study is to provide a novel dataset that enables researchers to explore and develop fusion methods that can be applied to larger datasets in the future.

It is worth mentioning that despite the participants’ diverse native languages, they were all fluent English speakers and used English regularly in their professional lives. Also, this aspect should have only a minor effect on the data, as a recent study by Malik-Moraleda, Saima, *et al*.^66^ has shown reported that “key properties of the neural architecture of language hold across speakers of diverse languages, and the variability observed across languages is similar to or lower than the inter-individual variability among speakers of the same language”.

## Data Availability

The code to preprocess the raw bimodal dataset as well as the two stimulation protocols (one for each modality) are publicly available at: https://github.com/LTU-Machine-Learning/Inner_Speech_EEG_FMRI.
